# A Retrospective Analysis of Extended Hospital Stays in Patients With Pneumonia and Diabetic Comorbidities in a Rural Midwestern Area

**DOI:** 10.7759/cureus.102915

**Published:** 2026-02-03

**Authors:** Shlok Rathi, Abbigail Niewchas, Max Balla, Brendan Lamboglia, Mariam Akhtar, Nova Beyersdorfer, Kerry Johnson, John Paulson

**Affiliations:** 1 Internal Medicine, Kansas City University of Medicine and Biosciences, Joplin, USA; 2 Family Medicine, Kansas City University of Medicine and Biosciences, Joplin, USA; 3 Primary Care, College of Medicine, Kansas City University, Joplin, USA; 4 Mathematics, Missouri Southern State University, Joplin, USA

**Keywords:** cardiovascular disease, diabetes mellitus, length of hospital stay (los), pneumonia

## Abstract

Pneumonia is one of the leading causes of hospitalization in the United States, resulting in many hospitalizations and deaths per year. Diabetes is an often-concurrent diagnosis that affects a large population. There is little research regarding the relationship between diabetes and the length of stay for patients hospitalized with pneumonia. This retrospective study aims to analyze whether the presence of diabetes mellitus in patients diagnosed with pneumonia increases the length of stay. Electronic medical records (EMR) from Freeman Health System in Southwest Missouri were utilized to analyze this relationship. Results of this study demonstrated that, in patients diagnosed with diabetes (type I or type II), pneumonia, or a combination of diabetes and pneumonia, pneumonia is the strongest predictor for a hospital stay ≥6 days. Further analysis, looking at age and gender, supported this finding, with pneumonia serving as the dominant factor in patients with hospital stays of ≥6 days duration. While there was no difference in the proportion of increased length of stay between age groups for combined pneumonia and diabetes, patients 65 years old and above had a larger proportion of extended length of stay compared to patients under 65 years old for both just pneumonia and just diabetes individually.

## Introduction

Diabetes Mellitus (DM) and pneumonia are both major contributors to healthcare spending and utilization, morbidity and mortality in the United States. DM, a chronic disorder of insulin production or resistance, affects tens of millions of Americans and was responsible for over 100,000 deaths in 2022 alone, ranking as the eighth leading cause of death nationally [1,2]. Beyond its direct complications on glucose regulation, DM increases the risk of infections, cardiovascular disease, renal impairment, and a wide range of other conditions, placing a substantial strain on healthcare resources.

Pneumonia, an infection of the lung parenchyma by bacteria, viruses, or fungi, accounts for over one million hospitalizations and over 50,000 deaths per year in the US [3,4]. Both DM and pneumonia have been individually associated with longer hospital stays and higher healthcare costs, but studies examining the combined effect of these conditions on the duration of inpatient stays are limited [5,6]. Furthermore, there is limited reporting on how patient age or gender may influence the length of stay in patients suffering from both conditions.

The purpose of this retrospective study is to evaluate whether the presence of DM in patients diagnosed with pneumonia is associated with an extended hospital stay, defined as ≥ 6 days, in a patient population from rural Southwest Missouri.

## Materials and methods

This retrospective study evaluated the impact of diabetes mellitus (DM), pneumonia, or both on the proportion of patients requiring an extended hospital stay, defined as ≥ 6 days. Electronic medical records (EMR) from Freeman Health System, encompassing two hospitals in southwest Missouri, were reviewed for admissions between January 1, 2019, and December 31, 2022. Patients were included if they were ≥ 18 years old at discharge and had at least one documented diagnosis listed in Tables 1-2. Only the first admission per patient was analyzed; patients with prior hospitalizations or without relevant diagnoses were excluded.

Patients were classified into three groups based on ICD-10 codes at admission: (1) PXG - pneumonia with DM, (2) P0G - pneumonia without DM, and (3) 0XG - DM without pneumonia. Admissions with pneumonia prior to the index admission were excluded.

The primary outcome was the proportion of patients in each group with a hospital stay ≥ 6 days. Subgroup analyses evaluated the effects of age (< 65 vs ≥ 65 years) and gender (male vs female) on extended hospitalization rates, with 95% confidence intervals (CIs) calculated for individual proportions and group comparisons. Comparisons of proportions between groups were performed using two-sample z-tests for proportions.

All statistical analyses were performed using Microsoft Excel (Microsoft Corp., Redmond, WA). P-values were reported to 15 decimal places and rounded to four decimal places for readability. All formulas utilized for the analysis were independently verified by the study statistician. Two-sample proportion z-tests were performed to compare group differences in the proportion of extended hospital stays. Statistical significance was defined as a two-tailed p-value < 0.05, with 95% confidence intervals reported for all estimates.

ICD-10 Codes Utilized in Screening Process

The ICD-10 codes that qualified patients as having met the inclusion criteria for a diagnosis of pneumonia or DM, respectively (Table 1-2).

**Table 1:** ICD-10 codes for pneumonia diagnoses.

ICD-10 Codes	Description
J1000	Influenza due to other identified influenza virus with unspecified type of pneumonia
J1001	Influenza due to other identified influenza virus with the same other identified influenza virus pneumonia
J1008	Influenza due to other identified influenza virus with other specified pneumonia
J1100	Influenza due to unidentified influenza virus with unspecified type of pneumonia
J1108	Influenza due to unidentified influenza virus with specified pneumonia
J120	Adenoviral pneumonia
J121	Respiratory syncytial virus pneumonia
J122	Parainfluenza virus pneumonia
J123	Human metapneumovirus pneumonia
J1281	Pneumonia due to SARS-associated coronavirus
J1282	Pneumonia due to coronavirus disease 2019
J1289	Other viral pneumonia
J129	Viral pneumonia, unspecified
J13	Pneumonia due to Streptococcus pneumoniae
J14	Pneumonia due to Hemophilus influenzae
J150	Pneumonia due to Klebsiella pneumoniae
J151	Pneumonia due to Pseudomonas
J1520	Pneumonia due to staphylococcus, unspecified
J15211	Pneumonia due to Methicillin susceptible Staphylococcus aureus
J15212	Pneumonia due to Methicillin resistant Staphylococcus aureus
J1529	Pneumonia due to other staphylococcus
J153	Pneumonia due to streptococcus, group B
J154	Pneumonia due to other streptococci
J155	Pneumonia due to Escherichia coli
J156	Pneumonia due to other Gram-negative bacteria
J157	Pneumonia due to Mycoplasma pneumoniae
J158	Pneumonia due to other specified bacteria
J159	Unspecified bacterial pneumonia
J160	Chlamydial pneumonia
J168	Pneumonia due to other specified infectious organisms
J17	Pneumonia in diseases classified elsewhere
J180	Bronchopneumonia, unspecified organism
J181	Lobar pneumonia, unspecified organism
J188	Other pneumonia, unspecified organism
J189	Pneumonia, unspecified organism
J84116	Cryptogenic organizing pneumonia
J851	Abscess of lung with pneumonia
J95851	Ventilator associated pneumonia

**Table 2:** ICD-10 codes for diabetes mellitus diagnoses.

ICD-10 Codes	Diagnosis
E0822	Diabetes mellitus due to underlying condition with diabetic chronic kidney disease
E0840	Diabetes mellitus due to underlying condition with diabetic neuropathy, unspecified
E08649	Diabetes mellitus due to underlying condition with hypoglycemia without coma
E0865	Diabetes mellitus due to underlying condition with hyperglycemia
E088	Diabetes mellitus due to underlying condition with unspecified complications
E089	Diabetes mellitus due to underlying condition without complications
E0922	Drug or chemical induced diabetes mellitus with diabetic chronic kidney disease
E0951	Drug or chemical induced diabetes mellitus with diabetic peripheral angiopathy without gangrene
E09649	Drug or chemical induced diabetes mellitus with hypoglycemia without coma
E0965	Drug or chemical induced diabetes mellitus with hyperglycemia
E098	Drug or chemical induced diabetes mellitus with unspecified complications
E099	Drug or chemical induced diabetes mellitus without complications
E1010	Type 1 diabetes mellitus with ketoacidosis without coma
E1011	Type 1 diabetes mellitus with ketoacidosis with coma
E1021	Type 1 diabetes mellitus with diabetic nephropathy
E1022	Type 1 diabetes mellitus with diabetic chronic kidney disease
E1029	Type 1 diabetes mellitus with other diabetic kidney complication
E10319	Type 1 diabetes mellitus with unspecified diabetic retinopathy without macular edema
E103493	Type 1 diabetes mellitus with severe nonproliferative diabetic retinopathy without macular edema, bilateral
E103592	Type 1 diabetes mellitus with proliferative diabetic retinopathy without macular edema, left eye
E103599	Type 1 diabetes mellitus with proliferative diabetic retinopathy without macular edema, unspecified eye
E1039	Type 1 diabetes mellitus with other diabetic ophthalmic complication
E1040	Type 1 diabetes mellitus with diabetic neuropathy, unspecified
E1042	Type 1 diabetes mellitus with diabetic polyneuropathy
E1043	Type 1 diabetes mellitus with diabetic autonomic (poly)neuropathy
E1051	Type 1 diabetes mellitus with diabetic peripheral angiopathy without gangrene
E1052	Type 1 diabetes mellitus with diabetic peripheral angiopathy with gangrene
E10610	Type 1 diabetes mellitus with diabetic neuropathic arthropathy
E10620	Type 1 diabetes mellitus with diabetic dermatitis
E10621	Type 1 diabetes mellitus with foot ulcer
E10622	Type 1 diabetes mellitus with other skin ulcer
E10628	Type 1 diabetes mellitus with other skin complications
E10649	Type 1 diabetes mellitus with hypoglycemia without coma
E1065	Type 1 diabetes mellitus with hyperglycemia
E1069	Type 1 diabetes mellitus with other specified complication
E108	Type 1 diabetes mellitus with unspecified complications
E109	Type 1 diabetes mellitus without complications
E1100	Type 2 diabetes mellitus with hyperosmolarity without nonketotic hyperglycemic-hyperosmolar coma (NKHHC)
E1101	Type 2 diabetes mellitus with hyperosmolarity with coma
E1110	Type 2 diabetes mellitus with ketoacidosis without coma
E1111	Type 2 diabetes mellitus with ketoacidosis with coma
E1121	Type 2 diabetes mellitus with diabetic nephropathy
E1122	Type 2 diabetes mellitus with diabetic chronic kidney disease
E1129	Type 2 diabetes mellitus with other diabetic kidney complication
E11311	Type 2 diabetes mellitus with unspecified diabetic retinopathy with macular edema
E11319	Type 2 diabetes mellitus with unspecified diabetic retinopathy without macular edema
E113299	Type 2 diabetes mellitus with mild nonproliferative diabetic retinopathy without macular edema, unspecified eye
E113549	Type 2 diabetes mellitus with proliferative diabetic retinopathy with combined traction retinal detachment and rhegmatogenous retinal detachment, unspecified eye
E113553	Type 2 diabetes mellitus with stable proliferative diabetic retinopathy, bilateral
E113593	Type 2 diabetes mellitus with proliferative diabetic retinopathy without macular edema, bilateral
E113599	Type 2 diabetes mellitus with proliferative diabetic retinopathy without macular edema, unspecified eye
E1136	Type 2 diabetes mellitus with diabetic cataract
E1139	Type 2 diabetes mellitus with other diabetic ophthalmic complication
E1140	Type 2 diabetes mellitus with diabetic neuropathy, unspecified
E1141	Type 2 diabetes mellitus with diabetic mononeuropathy
E1142	Type 2 diabetes mellitus with diabetic polyneuropathy
E1143	Type 2 diabetes mellitus with diabetic autonomic (poly)neuropathy
E1144	Type 2 diabetes mellitus with diabetic amyotrophy
E1149	Type 2 diabetes mellitus with other diabetic neurological complication
E1151	Type 2 diabetes mellitus with diabetic peripheral angiopathy without gangrene
E1152	Type 2 diabetes mellitus with diabetic peripheral angiopathy with gangrene
E1159	Type 2 diabetes mellitus with other circulatory complications
E11610	Type 2 diabetes mellitus with diabetic neuropathic arthropathy
E11618	Type 2 diabetes mellitus with other diabetic arthropathy
E11620	Type 2 diabetes mellitus with diabetic dermatitis
E11621	Type 2 diabetes mellitus with foot ulcer
E11622	Type 2 diabetes mellitus with other skin ulcer
E11628	Type 2 diabetes mellitus with other skin complications
E11641	Type 2 diabetes mellitus with hypoglycemia with coma
E11649	Type 2 diabetes mellitus with hypoglycemia without coma
E1165	Type 2 diabetes mellitus with hyperglycemia
E1169	Type 2 diabetes mellitus with other specified complication
E118	Type 2 diabetes mellitus with unspecified complications
E119	Type 2 diabetes mellitus without complications
E1310	Other specified diabetes mellitus with ketoacidosis without coma
E1321	Other specified diabetes mellitus with diabetic nephropathy
E1322	Other specified diabetes mellitus with diabetic chronic kidney disease
E13319	Other specified diabetes mellitus with unspecified diabetic retinopathy without macular edema
E1340	Other specified diabetes mellitus with diabetic neuropathy, unspecified
E1342	Other specified diabetes mellitus with diabetic polyneuropathy
E1343	Other specified diabetes mellitus with diabetic autonomic (poly)neuropathy
E1351	Other specified diabetes mellitus with diabetic peripheral angiopathy without gangrene
E1352	Other specified diabetes mellitus with diabetic peripheral angiopathy with gangrene
E13621	Other specified diabetes mellitus with foot ulcer
E13649	Other specified diabetes mellitus with hypoglycemia without coma
E1365	Other specified diabetes mellitus with hyperglycemia
E1369	Other specified diabetes mellitus with other specified complication
E139	Other specified diabetes mellitus without complications

## Results

Screening Processes

**Figure 1:**
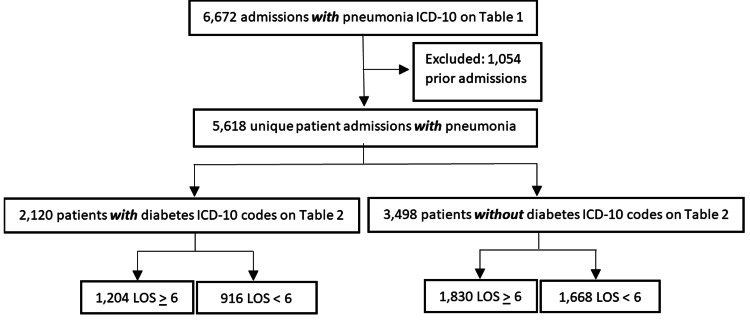
Flow chart demonstrating the inclusion and exclusion criteria for patients admitted with a diagnosis of pneumonia and with a diagnosis of diabetes.

To examine the relationship that a diagnosis of pneumonia, DM, or a combination of both has with the proportion of patients requiring an extended hospital stay, patient admissions data were first screened for an ICD-10 code for pneumonia. As demonstrated in Figure 1, 6,672 patient charts were found to have an ICD-10 code for pneumonia (Table 1) at the time of admission. Of these, 1,054 were determined to be a prior admission and were excluded from the analysis. The remaining 5,618 patient admissions were then screened for the presence of an ICD-10 code for DM. This search yielded 2,120 patient admissions with ICD-10 codes for both diseases (PXG group) and 3,498 patient admissions with a diagnosis of pneumonia that did not have a diagnosis of DM (P0G group).

**Figure 2:**
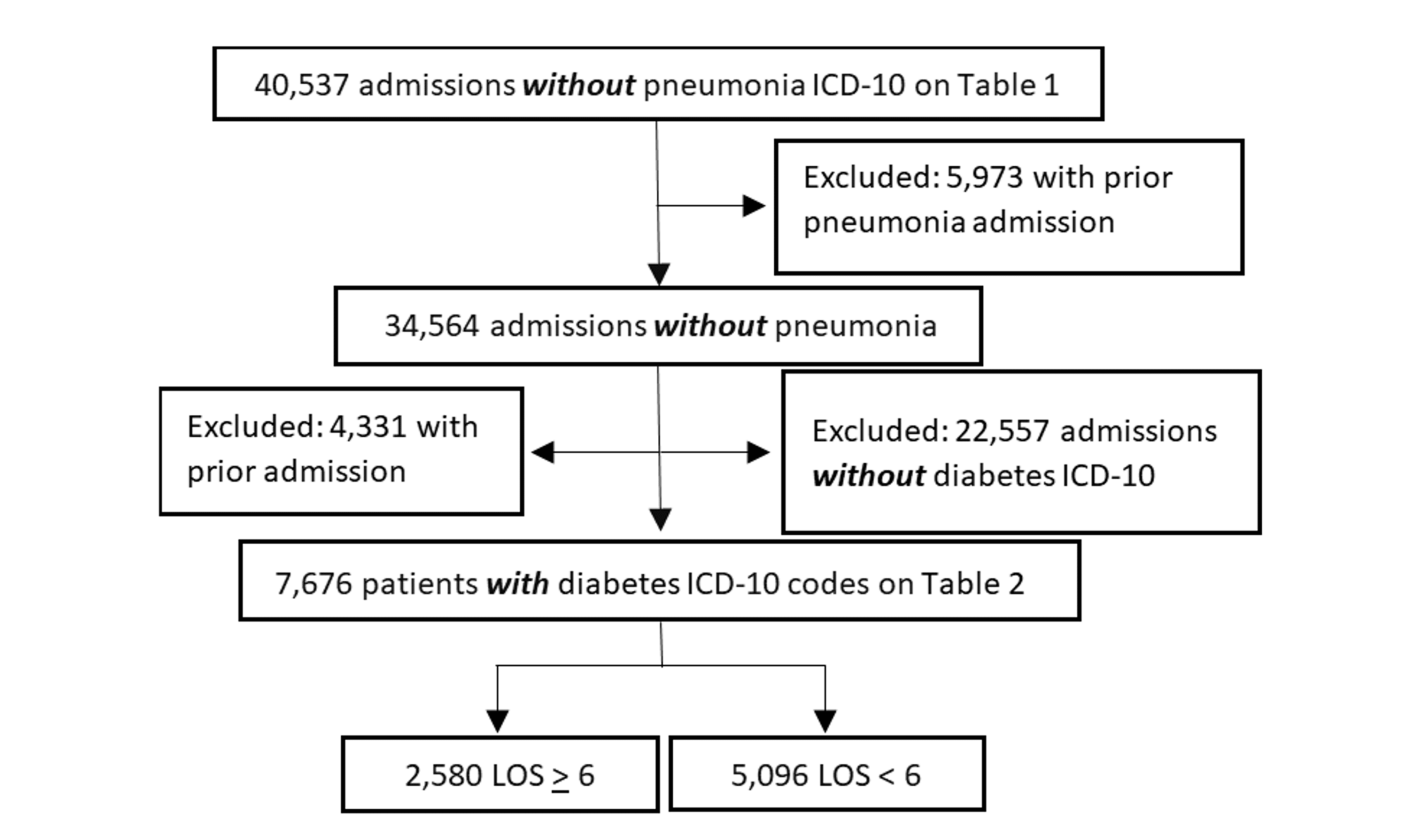
Flow chart demonstrating the inclusion and exclusion criteria for patients admitted without a diagnosis of pneumonia and with a diagnosis of diabetes.

Next, to investigate the relationship between the diagnosis of DM and the proportion of patients requiring an extended hospital stay, patient admissions without the presence of an ICD-10 code for pneumonia were analyzed for a subsequent ICD-10 code for DM. As shown in Figure 2, 40,537 admissions without an ICD-10 code for pneumonia were included in the initial screening. Of these, 5,973 were excluded for the presence of a prior admission for pneumonia. From the remaining 34,564 admissions, 22,557 were found to lack an ICD-10 code for DM, and 4,331 were prior hospital admissions and were also excluded. This yielded 7,676 patient admissions without an ICD-10 code for pneumonia, or prior admission for pneumonia, who had an ICD-10 code for DM (0XG group).

Analysis

As described in the previous section, patients were placed into one of three groups to determine the correlation of pneumonia, DM, or a combination of the two diagnoses on the proportion of patients requiring an extended hospital stay, defined as ≥6 days. A description and quantity of patients in each of these groups can be found in Table 3.

**Table 3:** Description and quantity of patients included in each group for initial analysis PXG: Pneumonia with DM; P0G: Pneumonia without DM; 0XG: DM without pneumonia; LOS: Length of stay

Groups	Description	Quantity
PXG	Pneumonia with diabetes (type I & II)	2,120
P0G	Pneumonia without diabetes (type I & II)	3,498
0XG	Diabetes (type I & II) without pneumonia	7,676

**Figure 3:**
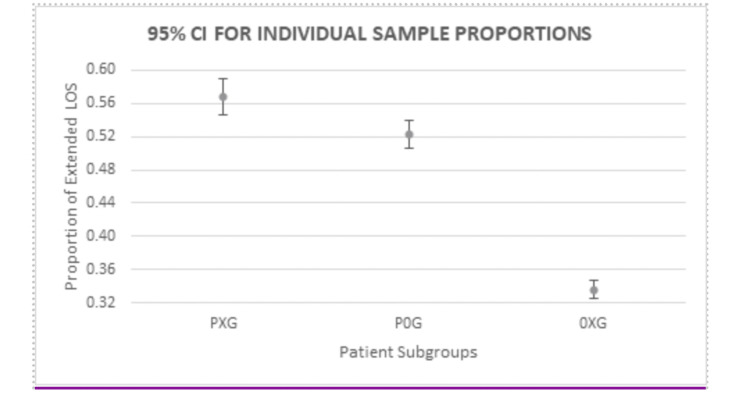
95% Confidence Intervals for the proportion of patients in each group requiring an extended stay. PXG: Pneumonia with DM; P0G: Pneumonia without DM; 0XG: DM without pneumonia; LOS: Length of stay

Individual sample proportions were compared to determine the number of patients in each group requiring an extended hospital stay. As demonstrated 1,204 of 2,120 patients in the PXG group (57%, 95% CI 55-59%) and 1,830 of 3,498 patients in the P0G group (52%, 95% CI 51-54%) required an extended stay, while 2,580 of 7,676 patients in the 0XG group (34%, 95% CI 33-35%) required an extended stay (Figure 3).

Two-sample proportion tests were then performed to assess the association between pneumonia and/or DM and the likelihood of an extended hospital stay. As shown in Table 4, the proportion of patients requiring an extended stay was slightly but significantly higher in the PXG group compared to the P0G group (95% CI 2-7%, p = 0.0011). Comparing the P0G group to the 0XG group, the difference in proportions was larger and also significant (95% CI 17-21%, p < 0.0001). Finally, the largest difference was observed between the PXG and 0XG groups, with a significantly higher percentage of PXG patients requiring an extended stay compared to 0XG patients (95% CI 21-26%, p < 0.0001).

**Table 4:** 95% confidence intervals for group comparisons. PXG: Pneumonia with DM; P0G: Pneumonia without DM; 0XG: DM without pneumonia; LOS: Length of stay

Comparison	LOS Sample 1	LOS Sample 2	Sample 1 vs Sample 2	Lower 95% CI for P1-P2	Upper 95% CI for P1-P2	Z-Test	P-value
PXG vs P0G	1204/2120	1830/3498	0.0448	0.0180	0.0716	3.2635	0.0011
	(0.5679)	(0.5232)					
PXG vs 0XG	1204/2120	2580/7676	0.2318	0.2082	0.2554	19.4049	<0.0001
	(0.5679)	(0.3361)					
P0G vs 0XG	1830/3498 (0.5232)	2580/7676 (0.3361)	0.1870	0.1674	0.2067	18.7587	<0.0001

Age Analysis

Of the 13,294 patients included in this review, 7,731 (58.15%) were elderly (defined as age ≥ 65 years old). An analysis was carried out to determine if age was a driving factor for increasing the proportion of patients requiring an extended hospital stay in the population analyzed. As demonstrated in Table 5, each group from the original analysis was further divided based on patient age. Subgroups ending with an ‘E’ represent the population of patients ≥ 65 years old in each group (elderly), while subgroups ending with ‘A’ represent the population of patients < 65 years old in each group (adult).

**Table 5:** Patient sub-grouping based on age.

Groups	
PXE	Pneumonia with diabetes (type I & II) ≥ 65
P0E	Pneumonia without diabetes (type I & II) ≥ 65
0XE	Diabetes (type I & II) without pneumonia ≥ 65
PXA	Pneumonia with diabetes (type I & II) < 65
P0A	Pneumonia without diabetes (type I & II) < 65
0XA	Diabetes (type I & II) without pneumonia < 65

**Figure 4:**
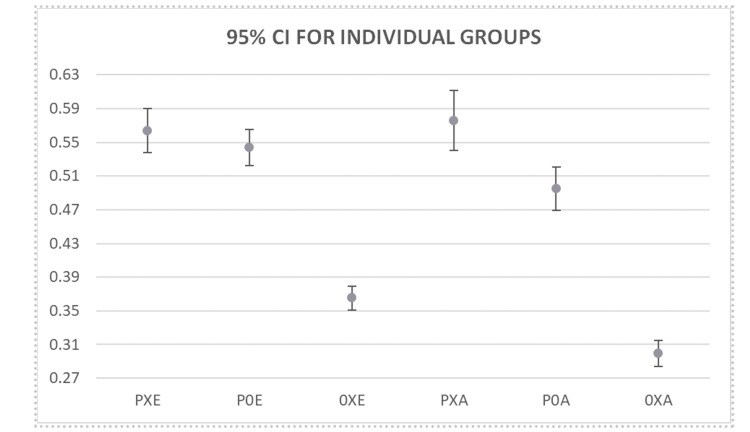
95% Confidence Intervals for the proportion of patients in each age group requiring an extended stay (E’’: Elderly subgroup, age ≥ 65 ‘A’ suffix: Adult subgroup, age < 65 years old). PXE: Pneumonia with diabetes (type I & II) ≥ 65; P0E: Pneumonia without diabetes (type I & II) ≥ 65; 0XE: Diabetes (type I & II) without pneumonia ≥ 65: PXA: Pneumonia with diabetes (type I & II) < 65; P0A: Pneumonia without diabetes (type I & II) < 65; 0XA: Diabetes (type I & II) without pneumonia < 65.

Individual sample proportions with 95% confidence intervals (CIs) for patients aged <65 years and ≥ 65 years in each group requiring an extended hospital stay are presented in Figure 4. Among patients aged < 65 years, 778 of 1,380 patients in the PXE group (56%, 95% CI 54-59%) and 1,102 of 2,027 patients in the P0E group (54%, 95% CI 52-57%) required an extended stay, while 1,578 of 4,324 patients in the 0XE group (36%, 95% CI 35-38%) did so. Among patients aged ≥ 65 years, 426 of 740 patients in the PXA group (58%, 95% CI 54-61%) and 728 of 1,471 patients in the P0A group (50%, 95% CI 47-52%) required an extended stay, whereas 1,002 of 3,352 patients in the 0XA group (30%, 95% CI 28-31%) did so. Overall, regardless of age, the presence of pneumonia was associated with a higher proportion of patients requiring an extended hospital stay compared with subgroups without pneumonia. 

Two-sample proportion tests were conducted to determine the effect of age on the proportion of patients requiring an extended hospital stay in each subgroup. Among patients aged < 65 years, 778 of 1,380 PXE patients (56%, 95% CI 54-59%) and 1,102 of 2,027 P0E patients (54%, 95% CI 52-57%) had a significantly higher proportion of extended stays compared to 1,578 of 4,324 0XE patients (36%, 95% CI 35-38%) (PXE vs 0XE: difference 20%, 95% CI 17-23%, p < 0.0001; P0E vs 0XE: difference 18%, 95% CI 15-20%, p < 0.0001). There was no significant difference between the PXE and P0E subgroups (difference 2%, p = 0.2466). 

Among patients aged ≥ 65 years, 426 of 740 PXA patients (58%, 95% CI 54-61%) and 728 of 1,471 P0A patients (50%, 95% CI 47-52%) had a significantly higher proportion of extended stays compared to 1,002 of 3,352 0XA patients (30%, 95% CI 28-31%) (PXA vs 0XA: difference 28%, 95% CI 24-32%, p < 0.0001; P0A vs 0XA: difference 20%, 95% CI 17-23%, p < 0.0001). Additionally, a small but statistically significant difference was observed between PXA and P0A (difference 8%, 95% CI 4-12%, p = 0.0003). 

Comparisons across age groups were also performed. PXE (age ≥ 65, pneumonia with DM) vs PXA (age < 65, pneumonia with DM) showed no significant difference (difference 1%, p = 0.5978). PXE vs P0A (age < 65, pneumonia without DM) showed a small but significant difference (7%, 95% CI 3-11%, p = 0.0002), and PXE vs 0XA (age < 65, DM without pneumonia) showed a larger significant difference (26%, 95% CI 23-30%, p < 0.0001). 

For patients with pneumonia without DM ≥ 65 (P0E), comparisons to other subgroups showed no significant difference with PXA (difference 3%, p = 0.1339), but a small yet significant difference compared to P0A (difference 5%, 95% CI 2-8%, p = 0.0044) and a larger significant difference compared to 0XA (difference 24%, 95% CI 22-27%, p < 0.0001). 

Finally, for patients with DM without pneumonia ≥ 65 (0XE), significantly higher proportions of extended stays were observed when compared to PXA, P0A, and 0XA subgroups (differences 21%, 95% CI 17-25%; 13%, 95% CI 10-16%; 7%, 95% CI 5-9%; p < 0.0001 for all). A summary of all subgroup comparisons is provided in Table 6. 

**Table 6:** 95% Confidence Intervals for group comparisons (‘-’ indicates that no significant difference was detected; therefore, population parameters may or may not be the same.) PXE: Pneumonia with diabetes (type I & II) ≥ 65; P0E: Pneumonia without diabetes (type I & II) ≥ 65; 0XE: Diabetes (type I & II) without pneumonia ≥ 65: PXA: Pneumonia with diabetes (type I & II) < 65; P0A: Pneumonia without diabetes (type I & II) < 65; 0XA: Diabetes (type I & II) without pneumonia < 65.

Comparison	LOS Sample 1	LOS Sample 2	Sample 1 vs Sample 2	Lower 95% CI for S1-S2	Upper 95% CI for S1-S2	Z-Test	P-value
PXE vs P0E	778/1380	1102/2027	0.0201	-	-	1.1585	0.2466
	(0.5638)	(0.5437)					
PXE vs 0XE	778/1380	1578/4324	0.1988	0.1690	0.2287	13.0608	<0.0001
	(0.5638)	(0.3649)					
PXE vs PXA	778/1380	426/740	0.0119	-	-	0.5276	0.5978
	(0.5638)	(0.5757)					
PXE vs P0A	778/1380	728/1471	0.0689	0.0323	0.1054	3.6811	0.0002
	(0.5638)	(0.4949)					
PXE vs 0XA	778/1380	1002/3352	0.2648	0.2344	0.2953	17.0936	<0.0001
	(0.5638)	(0.2989)					
P0E vs 0XE	1102/2027	1578/4324	0.1787	0.1527	0.2047	13.4433	<0.0001
	(0.5437)	(0.3649)					
P0E vs PXA	1102/2027	426/740	0.0320	-	-	1.4990	0.1339
	(0.5437)	(0.5757)					
P0E vs P0A	1102/2027	728/1471	0.0488	0.0152	0.0823	2.8502	0.0044
	(0.5437)	(0.4949)					
P0E vs 0XA	1102/2027	1002/3352	0.2447	0.2181	0.2714	17.8236	<0.0001
	(0.5437)	(0.2989)					
0XE vs PXA	1578/4324	426/740	0.2107	0.1723	0.2491	10.8326	<0.0001
	(0.3649)	(0.5757)					
0XE vs P0A	1578/4324	728/1471	0.1300	0.1007	0.1593	8.7965	<0.0001
	(0.3649)	(0.4949)					
0XE vs 0XA	1578/4324	1002/3352	0.0660	0.0449	0.0871	6.0726	<0.0001
	(0.3649)	(0.2989)					
PXA vs P0A	426/740	728/1471	0.0808	0.0369	0.1246	3.5880	0.0003
	(0.5757)	(0.4949)					
PXA vs 0XA	426/740	1002/3352	0.2767	0.2379	0.3156	14.2952	<0.0001
	(0.5757)	(0.2989)					
P0A vs 0XA	728/1471	1002/3352	0.1960	0.1661	0.2259	13.0649	<0.0001

Gender Analysis

The final variable analyzed for an association with an increased proportion of patients requiring an extended stay was patient gender. Of the patient population utilized for this analysis, 53.23% were male, and 46.77% were female. Table 7 outlines the subgroup designations when patients were further divided based on gender.

**Table 7:** Patient sub-grouping based on gender.

Groups	
PXM	Pneumonia with diabetes (type I & II) male
P0M	Pneumonia without diabetes (type I & II) male
0XM	Diabetes (type I & II) without pneumonia male
PXF	Pneumonia with diabetes (type I & II) female
P0F	Pneumonia without diabetes (type I & II) female
0XF	Diabetes (type I & II) without pneumonia female

**Figure 5:**
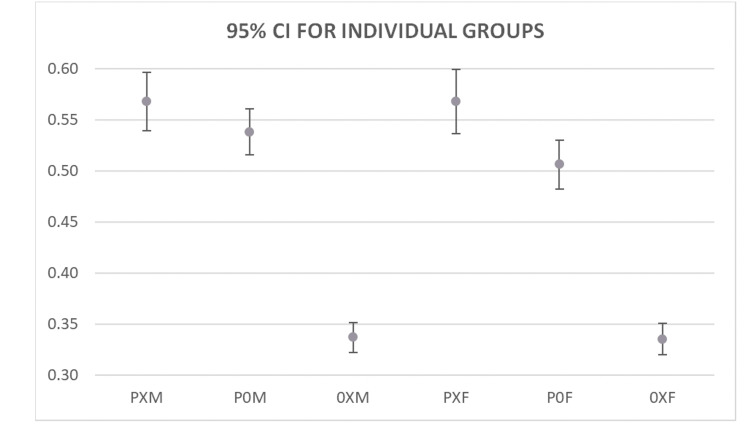
95% Confidence Intervals for individual groups based on gender. PXM: Pneumonia with diabetes (type I & II) male; P0M: Pneumonia without diabetes (type I & II) male; 0XM Diabetes (type I & II) without pneumonia male; PXF: Pneumonia with diabetes (type I & II) female; P0F: Pneumonia without diabetes (type I & II) female; 0XF: Diabetes (type I & II) without pneumonia female

Individual sample proportions with 95% confidence intervals (CIs) were also calculated for male and female patients in each group requiring an extended hospital stay. Among male patients, 664 of 1,169 PXM patients (57%, 95% CI 54-60%) and 987 of 1,833 P0M patients (54%, 95% CI 52-56%) required an extended stay, compared to 1,373 of 4,075 0XM patients (34%, 95% CI 32-35%). Among female patients, 540 of 951 PXF patients (57%, 95% CI 54-60%) and 843 of 1,665 P0F patients (51%, 95% CI 48-53%) required an extended stay, compared to 1,207 of 3,601 0XF patients (34%, 95% CI 32-35%). Overall, pneumonia was associated with a higher proportion of patients requiring extended hospitalization regardless of gender. A summary of these findings is demonstrated in Figure 5. 

Two-sample proportion tests were conducted to evaluate gender-specific differences. Among males, both PXM and P0M subgroups had a significantly higher proportion of extended stays compared to 0XM (PXM vs 0XM: difference 23%, 95% CI 20-26%, p < 0.0001; P0M vs 0XM: difference 20%, 95% CI 17-23%, p < 0.0001), while no significant difference was observed between PXM and P0M (difference 3%, p = 0.1126). Among females, both PXF and P0F had a significantly higher proportion of extended stays compared to 0XF (PXF vs 0XF: difference 23%, 95% CI 20-27%, p < 0.0001; P0F vs 0XF: difference 17%, 95% CI 14-20%, p < 0.0001). Unlike males, a small but significant difference was observed between PXF and P0F (difference 6%, 95% CI 2-10%, p = 0.0024). 

Comparisons across genders revealed no significant difference between PXM and PXF (difference 0%, p = 0.9932). However, PXM had a slightly higher proportion of extended stays compared to P0F (difference 6%, 95% CI 2-10%, p = 0.0012) and a significantly higher proportion compared to 0XF (difference 23%, 95% CI 20-27%, p < 0.0001). P0M did not differ significantly from PXF or P0F (differences 3%, p = 0.1398 and 3%, p = 0.0572, respectively) but had a significantly higher proportion compared to 0XF (difference 20%, 95% CI 18-23%, p < 0.0001). 

Finally, 0XM had significantly higher proportions of extended stays compared to PXF and P0F (differences 23%, 95% CI 20-27% and 17%, 95% CI 14-20%, p < 0.0001 for both), but no significant difference was observed compared to 0XF (difference 0%, p = 0.8715). A summary of all gender-based comparisons is provided in Table 8. 

**Table 8:** 95% Confidence Intervals for group comparisons based on gender (‘-’ indicates that no significant difference was detected; therefore, population parameters may or may not be the same.) PXM: Pneumonia with diabetes (type I & II) male; P0M: Pneumonia without diabetes (type I & II) male; 0XM Diabetes (type I & II) without pneumonia male; PXF: Pneumonia with diabetes (type I & II) female; P0F: Pneumonia without diabetes (type I & II) female; 0XF: Diabetes (type I & II) without pneumonia female

Comparison	LOS Sample 1	LOS Sample 2	Sample 1 vs Sample 2	Lower 95% CI for S1-S2	Upper 95% CI for S1-S2	Z-Test	P-value
PXM vs P0M	664/1169	987/1833	0.0295	-	-	1.5866	0.1126
	(0.5680)	(0.5385)					
PXM vs 0XM	664/1169	1373/4075	0.2311	0.1992	0.2630	14.2892	<0.0001
	(0.5680)	(0.3369)					
PXM vs PXF	664/1169	540/951	0.0002	-	-	0.0085	0.9932
	(0.5680)	(0.5678)					
PXM vs P0F	664/1169	843/1665	0.0617	0.0245	0.0989	3.2405	0.0012
	(0.5680)	(0.5063)					
PXM vs 0XF	664/1169	1207/3601	0.2328	0.2005	0.2651	14.1658	<0.0001
	(0.5680)	(0.3352)					
P0M vs 0XM	987/1833	1373/4075	0.2015	0.1745	0.2286	14.6304	<0.0001
	(0.5385)	(0.3369)					
P0M vs PXF	987/1833	540/951	0.0294	-	-	1.4764	0.1398
	(0.5385)	(0.5678)					
P0M vs P0F	987/1833	843/1665	0.0322	-	-	1.9016	0.0572
	(0.5385)	(0.5063)					
P0M vs 0XF	987/1833	1207/3601	0.2033	0.1757	0.2308	14.4394	<0.0001
	(0.5385)	(0.3352)					
0XM vs PXF	1373/4075	540/951	0.2309	0.1962	0.2656	13.2046	<0.0001
	(0.3369)	(0.5678)					
0XM vs P0F	1373/4075	843/1665	0.1694	0.1413	0.1974	11.9611	<0.0001
	(0.3369)	(0.5063)					
0XM vs 0XF	1373/4075	1207/3601	0.0017	-	-	0.1618	0.8715
	(0.3369)	(0.3352)					
PXF vs P0F	540/951	843/1665	0.0615	0.0219	0.1011	3.0319	0.0024
	(0.5678)	(0.5063)					
PXF vs 0XF	540/951	1207/3601	0.2326	0.1976	0.2677	13.1212	<0.0001
	(0.5678)	(0.3352)					
P0F vs 0XF	843/1665	1207/3601	0.1711	0.1426	0.1997	11.8421	<0.0001
	(0.5063)	(0.3352)					

## Discussion

This study demonstrates that the coexistence of diabetes mellitus (DM) and pneumonia is associated with a higher likelihood of prolonged hospitalization (greater than or equal to six days) when compared with either condition alone. Patients with both DM and pneumonia had a 56.79% probability of requiring an extended length of stay, compared with 52.23% for pneumonia alone and 33.61% for DM alone. These findings align with prior literature showing that both conditions independently contribute to increased duration of hospitalization and increased risk of complications, suggesting a synergistic effect in patients with both diagnoses.

Previous studies have demonstrated that pneumonia is a stronger driver of prolonged hospitalization compared to DM alone. Bader et al. reported that patients hospitalized with community-acquired pneumonia (CAP) experienced significantly longer lengths of stay in comparison to those admitted for DM without pneumonia, highlighting pneumonia as the primary determinant of hospitalization duration [7]. In contrast, Valent et al. showed that DM is independently associated with increased length of stay and in-hospital mortality, even in the absence of pneumonia, underscoring its role as a meaningful comorbidity rather than a dominant driver [8]. Subsequent cohort studies have further reported that patients with both DM and pneumonia experience longer hospitalizations than those with pneumonia alone, suggesting a synergistic effect on disease severity and recovery [9-11]. Notably, many of these prior analyses did not adjust for age or gender, variables that are known to influence hospitalization patterns and overall outcomes, limiting direct comparisons across patient subgroups and motivating the stratified approach used in this analysis.

This study extends this literature by explicitly examining age and gender as both independent variables and in combination with disease status. When stratified by age, patients aged 65 years or older with either DM or pneumonia alone demonstrated a higher proportion of extended hospital stays, consistent with prior reporting that advanced age is a determinant of pneumonia severity and recovery [11]. However, in patients with both DM and pneumonia, the proportion of extended stays was similar across age groups, suggesting that the combined disease burden may attenuate age-related differences in hospitalization duration. In contrast, no statistically significant differences in the proportion of patients requiring an extended stay were observed between genders, either as a standalone variable or when stratified by disease status.

Consistent with prior observational studies, pneumonia emerged as the strongest predictor of extended hospitalization, with 52.23% of patients experiencing a length of stay greater than or equal to six days compared with 33.61% of patients with DM alone. However, the presence of concomitant DM significantly increased the proportion of extended stays among patients with pneumonia to 56.79%, supporting the notion of a synergistic effect. These findings align with broader consensus data demonstrating modest but clinically meaningful increases in length of stay among patients hospitalized with pneumonia, potentially mediated by metabolic dysregulation and impaired immune responses.

This study has several strengths, including a large sample size, the use of real-world clinical data from multiple hospitals, and stratified analyses by age and gender that provide additional context for interpreting the relationship between pneumonia, diabetes, and extended duration of hospital stay.

This study has several limitations. First, we did not differentiate between type I DM and type II DM or assess glycemic control using POC glucose trends or hemoglobin A1c, both of which have been associated with pneumonia outcomes [12,13]. Additionally, potential confounders, such as obesity, were not included and may independently influence hospitalization duration. Due to the retrospective nature of the study, informed consent was not required for the analysis of previously attained patient data, and the sample was not chosen at random. It is unable to be determined if the sample analyzed is representative of the general population. Future studies incorporating these variables in a broader population may further clarify the mechanisms underlying prolonged length of stay in this patient population. Finally, the statistical analyses relied on two-sample proportion tests, which assume independence of observations and adequate sample sizes for normal approximation. Although the large cohort supports these assumptions, unmeasured confounders inherent to retrospective datasets may still influence the observed associations.

## Conclusions

Overall, this study demonstrates that pneumonia is a stronger driver of prolonged hospitalization than DM alone, but that the coexistence of DM significantly increases the proportion of patients requiring an extended length of stay, regardless of age or gender. These findings are consistent with existing literature showing that DM modestly but meaningfully prolongs hospitalization in patients with pneumonia and may compound disease severity, potentially through impaired metabolic and immunologic mechanisms. Future studies incorporating DM subtype, glycemic control metrics, and additional comorbidities such as obesity may further clarify the pathways through which DM influences hospital length of stay and patient outcomes.
